# A Framework of Rebalancing Imbalanced Healthcare Data for Rare Events' Classification: A Case of Look-Alike Sound-Alike Mix-Up Incident Detection

**DOI:** 10.1155/2018/6275435

**Published:** 2018-05-22

**Authors:** Yang Zhao, Zoie Shui-Yee Wong, Kwok Leung Tsui

**Affiliations:** ^1^Department of Systems Engineering and Engineering Management, City University of Hong Kong, Kowloon, Hong Kong; ^2^Graduate School of Public Health, St. Luke's International University, Tokyo, Japan

## Abstract

Identifying rare but significant healthcare events in massive unstructured datasets has become a common task in healthcare data analytics. However, imbalanced class distribution in many practical datasets greatly hampers the detection of rare events, as most classification methods implicitly assume an equal occurrence of classes and are designed to maximize the overall classification accuracy. In this study, we develop a framework for learning healthcare data with imbalanced distribution via incorporating different rebalancing strategies. The evaluation results showed that the developed framework can significantly improve the detection accuracy of medical incidents due to look-alike sound-alike (LASA) mix-ups. Specifically, logistic regression combined with the synthetic minority oversampling technique (SMOTE) produces the best detection results, with a significant 45.3% increase in recall (recall = 75.7%) compared with pure logistic regression (recall = 52.1%).

## 1. Introduction

The rapid growth of electronic health records (EHRs) is generating massive health informatics and bioinformatics datasets, and more and more crowdsourced medical data are becoming available. Using statistical data analytics to detect rare but significant healthcare events in these massive unstructured dataset, such as medication errors and disease risk, has the potential to reduce treatment costs, avoid preventable diseases, and improve care quality in general [1, 2]. One major challenge to effective healthcare data analytics is highly skewed data class distribution, which is referred to as the imbalanced classification problem. An imbalanced classification problem occurs when the classes in a dataset have a highly unequal number of samples. For example, in a binary classification, the imbalanced classification problem is present when one class has significantly fewer observations than the other class. The former is usually called a minority class, and the latter, a majority class. In this study, we develop a method for detecting relevant healthcare events in datasets where this data challenge is present.

In some healthcare-related datasets with imbalanced classification, accurately detecting minority class observations is of great importance, as they correspond to high-impact events. For instance, some attempts have been made to automatically identify medical incident reports. The targeted medical incident reports are usually reports of incidents that have been recognized as common causes of medication errors that may result in adverse or harmful patient outcomes [3]. In practice, many datasets of medical incident reports exhibit imbalanced class distribution. For example, in an investigation of the classification of two types of medical incident reports, namely, “clinical management/inadequate handover” and “clinical management/incorrect patient,” Ong (2010) [4] found that there were more than twice as many clinical management/incorrect patient cases as clinical management/inadequate handover cases. In another example, Wong (2016) [5] examined the detection of look-alike and sound-alike (LASA) mix-up cases from the medical incident reports and found that only 21% of the available reports were related to LASA cases.

Conventional statistical learning classifiers typically perform poorly in imbalanced datasets, as they implicitly assume an equal occurrence of all classes and are designed to maximize the overall classification accuracy. Thus, these classifiers favor the majority class, resulting in poor accuracy in detecting minority class observations [6, 7]. Many healthcare data analytics applications have neglected the problem of dataset imbalance [8], and the effectiveness of classifiers that use rebalancing strategies to address the detection problem has rarely been evaluated [9, 10].

Resampling and cost-sensitive learning are state-of-the-art rebalancing strategies for imbalanced classification. The resampling schemes include randomly oversampling the minority class, undersampling the majority class, and some advanced synthetic sampling methods that attempt to rebalance class distribution at the data level. However, these rebalancing strategies have some limitations. For instance, an unavoidable consequence of undersampling is the loss of information [11], whereas oversampling through the random replication of the minority class sample usually creates very specific rules, leading to overfitting [7]. Cost-sensitive learning considers the costs of misclassified instances and minimizes the total misclassification cost, attempting to rebalance class distribution at the algorithm level. As cost-sensitive learning methods are motivated by the observation that most real applications do not have a unified cost for misclassification, the cost matrix needs be manually determined beforehand.

In this study, we develop a framework for analyzing healthcare data with imbalanced distribution that incorporates different rebalancing strategies, and we offer guidelines for choosing appropriate procedures. This learning framework consists of two main stages: selecting base classifiers and evaluating rebalancing strategies. We examine the effect of data imbalance on classifier performance and the effectiveness of various rebalancing strategies. The results of our analysis of a published study's dataset show that the developed framework significantly improves the accuracy in detecting medical incidents caused by LASA mix-ups. It is worth noting that the framework has a broad range of applications beyond medical incident reports detection, in datasets with similar imbalanced data properties.

## 2. Background

### 2.1. Imbalanced Data in Healthcare

**Figure 1:**
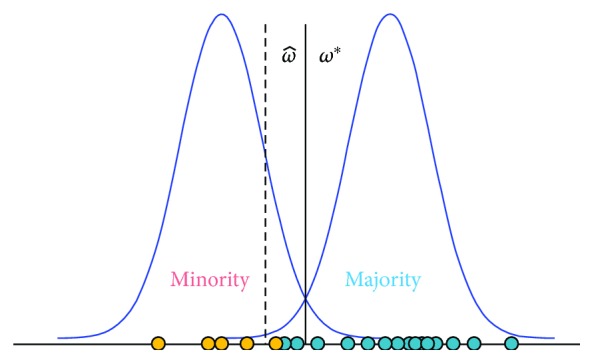
Bias of a linear separator.

The imbalance property that is common to many real healthcare datasets makes classification a challenging task. The imbalanced classification problem in the healthcare domain, where data are often highly skewed due to individual heterogeneity and diversity, affects issues such as cancer diagnostics [12, 13], patient safety informatics [5, 14], and disease risk prediction [15]. Most standard classifiers, such as logistic regression and the support vector machine, implicitly assume that both classes are equally common. Additionally, these methods are designed for maximizing overall classification accuracy. As a result, they favor the majority class, resulting in poor sensitivity toward the minority class [6, 7]. This intuition is illustrated in Figure 1, which contains a synthetic example containing a majority class and a minority class. The solid line (*ω*^∗^) depicts the optimal separator in the underlying distribution, and the dotted line ($\hat{\omega }$) is the max-margin loss-minimizing separator generated over the instances. In this case, the induced separator is clearly skewed toward the minority class.

The fundamental concern raised by the imbalanced learning problem is that the performance of most standard learning algorithms is significantly compromised by imbalanced data. Resampling at the data level and cost-sensitive learning at the algorithm level are common strategies for addressing imbalanced classification. In the following sections, we review these strategies and examine their effectiveness when they are combined with standard classifiers to detect medical incidents in imbalanced datasets.

### 2.2. Rebalancing Strategies

#### 2.2.1. Data-Level Approaches

Approaches at the data level, based on the observation that classifiers learn better from a balanced distribution than from an imbalanced one, use various methods for rebalancing the class distribution [4, 16]. The representative scheme is randomly oversampling the minority class and undersampling the majority class [17, 18] or a combination of both schemes. Some advanced methods, called synthetic sampling strategies, generate synthetic instances to improve classifiers' performance.

*(1) Oversampling and Undersampling*. Due to their simplicity and computational efficiency, over- and undersampling methodologies are popular strategies for countering the effect of imbalanced datasets [19–24]. The oversampling technique consists of randomly selecting instances from the minority class with replication and then adding the replications into the minority class. In this way, the size of the minority class is enlarged. Let **X** = {*x*_*i*_} (*i* = 1, 2,…, *N*) denote the minority class with *N* instances. Then, the randomly oversampled minority class is **X**_**o**_ = {*x*_*j*_} (*j* = 1, 2,…, *N*_*o*_), where ∀*x*_*j*_ ∈ **X** and *N*_*o*_ > *N*. In contrast, the undersampling scheme randomly removes instances from the majority class, which can also rebalance the minority and majority classes. Let **Z** = {**z**_**i**_} (*i* = 1, 2,…, *M*) denote the majority class with *M* instances. Then, the randomly undersampled majority class is **Z**_**o**_ = {*z*_*j*_} (*j* = 1, 2,…, *M*_*u*_), where ∀*z*_*j*_ ∈ **Z** and *M*_*u*_ < *M*. The random under- and oversampling methods each have shortcomings. An unavoidable drawback of undersampling is the loss of information [11], whereas oversampling, through the random replication of the minority class sample, usually creates very specific rules, leading to model overfitting [7].

*(2) Synthetic Sampling*. The synthetic minority oversampling technique (SMOTE) [25] is a typical synthetic sampling method that has been very successful in various applications and been the foundation for many variants of the basic synthetic sampling scheme [26]. SMOTE searches 𝑘-nearest minority neighbors of each minority instance (denoted as *x*_*i*_) and then randomly selects one of the neighbors as the reference point. The synthetic instance is generated by first multiplying the difference between the feature vector of the selected neighbor and *x*_*i*_ with a random value within the range [0,1]. Then, the following vector is added to *x*_*i*_:

(1) $$\begin{array}{c} x_{new}=x_{i}+\left({\hat{x}}_{i}-x_{i}\right)\times \delta , \end{array}$$

where ${\hat{x}}_{i}$ is one of the 𝑘-nearest neighbors for *x*_*i*_ and *δ* ∈ [0, 1] is a random number. In the implementation of SMOTE, there are two key parameters for controlling the amount of oversampling of the minority class and undersampling of the majority classes, that is, *α* and *γ*. For each case belonging to the minority class in the original dataset, *α*/100 new minority samples will be generated. The parameter *γ* controls the proportion of cases of the majority class that will be randomly selected for the final “balanced” dataset. This proportion is calculated with respect to the number of newly generated minority class cases. One potential drawback of SMOTE is that it generates the same number of synthetic data samples for each original minority example without considering neighboring examples, which may increase the occurrence of overlaps between classes [26].

#### 2.2.2. Algorithm-Level Approaches

Instead of rebalancing the class distribution at the data level, some solutions have been based on biasing the existing classifiers at the algorithm level. One popular approach is to use a cost-sensitive learning method [27, 28], which considers the costs of misclassified instances and minimizes the total misclassification cost.

*(1) Cost-Sensitive Learning*. Unlike the rebalancing strategy, cost-sensitive learning does not directly create a balanced class distribution. Instead, it highlights the imbalanced learning problem using a cost matrix that describes the cost of misclassification in a particular scenario. Cost-sensitive learning is motivated by the observation that most real applications do not have a unified cost for misclassification [28]; therefore, the cost matrix needs to be determined beforehand, which is a major limitation. In other words, this method evaluates the cost associated with misclassifying observations [29–32]. An illustration of a cost matrix can be found in Table 1, where C(FN) and C(FP) correspond to the costs associated with a false negative (FN) and a false positive (FP), respectively. Specifically, C(FN) > C(FP) defines an imbalanced classification.

The goal of cost-sensitive learning is to choose the classifier with the lowest total cost, that is,

(2) $$\begin{array}{c} Total\;cost=C\left(FN\right)\times FN+C\left(FP\right)\times FP. \end{array}$$

It is worth noting that if a parametric model-based classifier (e.g., logistic regression) is applied, then choosing a classifier with the lowest total cost can be done by varying the threshold based on the loss function (cost of false negatives to false positives) in the training data; this is the empirical thresholding method [33]. For example, in a logistic regression, let **x** ∈ ℝ^*d*^ denote the *d-*dimensional vector of explanatory variables and let *y* ∈ *Y* be the corresponding binary response (1 for minority and 0 for majority). The basic form of the posterior probability estimated via a linear function in **x** is as follows:

(3) $$\begin{array}{c} \mathrm{Pr}\left(y=1\;|\;x=x\right)=\frac{\exp\left(\beta_{0}+\beta^{T}x\right)}{1+\exp\left(\beta_{0}+\beta^{T}x\right)};\\ \mathrm{Pr}\left(y=0\;|\;x=x\right)=\frac{1}{1+\exp\left(\beta_{0}+\beta^{T}x\right)}. \end{array}$$

Given the monotone transformation, we have the following:

(4) $$\begin{array}{c} \log\frac{\mathrm{Pr}\left(y=1\;|\;x=x\right)}{\mathrm{Pr}\left(y=0\;|\;x=x\right)}=\beta_{0}+\beta^{T}x. \end{array}$$

The predicted log ratio is a hyperplane defined by $\left\{x|{\hat{\beta }}_{0}+{\hat{\beta }}^{T}x=\theta \right\}$, where *θ* is the tuning threshold for choosing a classifier with the lowest total cost.

All these methods deal with imbalanced classification by directly or indirectly rebalancing the class distribution in a dataset. Various studies have presented (sometimes conflicting) viewpoints on the usefulness of different rebalancing strategies, and a comprehensive discussion can be found in [20, 26, 34].

### 2.3. Framework for Learning Data with Imbalanced Distribution Using Rebalancing Strategies

**Figure 2:**
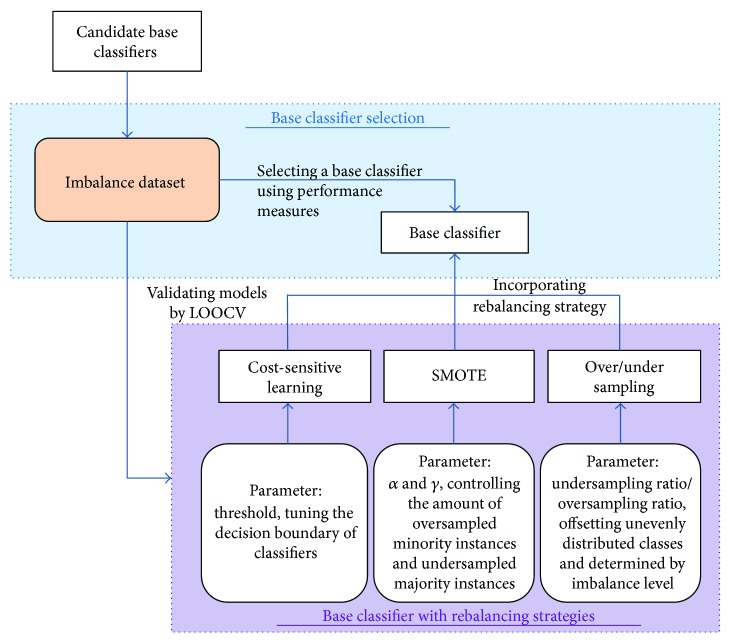
Framework of the learning procedure.

In this study, we develop a framework for learning healthcare data with imbalanced distribution by incorporating different rebalancing strategies and offering guideline procedure.  Figure 2 shows the framework for the entire learning procedure. This framework consists of two stages: selecting a base classifier and using the base classifier to implement rebalancing strategies. In the first stage, a base classifier is selected from a set of candidates by evaluating each classifier's performance metrics (e.g., recall can be used to evaluate whether the training classifier has correctly classified minority instances). The candidate classifiers can be either linear or nonlinear methods for binary classification (e.g., logistic regression (LR), support vector machine, decision tree, and linear discriminant analysis). In the second stage, rebalancing strategies (including resampling and cost-sensitive learning methods) can be combined with the base classifier and implemented across a range of parameters. The possible parameter sets must be designed based on the guidelines for designing the parameter/threshold of each rebalancing strategy (e.g., the oversampling ratio for an oversampling strategy should be determined based on the imbalance level). Leave-one-out cross-validation (LOOCV), which is a fair way to properly estimate model prediction performance, is used to evaluate the performance of classifiers in these stages.

## 3. Materials and Methods

We carry out a set of experiments to verify the effectiveness of our developed framework using a practical healthcare imbalanced dataset. We present the selected imbalanced binary classification problem, our experimental design, and the evaluation criteria used in this study as follows.

### 3.1. Case Description: LASA Cases

Medication names that look alike and sound alike have been recognized as the most common cause of medication errors [3]. Furthermore, 1.4% of errors due to LASA drug mix-ups have resulted in adverse and harmful patient outcomes [35, 36]. The timely and accurate identification of medication errors due to LASA drug mix-ups would reduce the medical risk to patients. Wong (2016) [5] used GPSA medical incident reports to construct classifiers for detecting LASA cases and acknowledged the challenges arising from the imbalanced classification of patient safety incident data. In our experiments, we evaluate our proposed imbalanced classification framework for detecting LASA cases using Wong's dataset [5]. The raw dataset is unstructured, as the medical incident reports are in free text format. The structured dataset used in the subsequent modeling and evaluation is a 227 × 8 dataset with 48 minority cases and 179 majority cases [5]. We thus regard LASA and non-LASA cases as minority and majority classes, respectively.

### 3.2. Base Classifiers

We compare the performance of several conventional classifiers, including the logistic regression (LR) [37], support vector machine with linear kernel (L.SVM), support vector machine with radial kernels (R.SVM) [38], and decision tree (DT) [39]. Many healthcare applications have used these classifiers due to their simplicity, interpretability, and computation efficiency [4, 5, 40]. In this study, we directly apply and validate these methods on the dataset and select the most effective classifier for detecting LASA cases as the base classifier. This base classifier is then combined with several rebalancing strategies in the second stage of the study.

### 3.3. Experiment Design

We investigate the performance of classifiers under various rebalancing strategies, including oversampling, undersampling, SMOTE, and cost-sensitive learning. As parameter settings usually have a significant impact on a classifier's performance, they are thoroughly assessed in the implementation. 1) For oversampling, the oversampling ratio (number of oversampled minority instances/Number of minority instances) is set within the interval [1, 5] (*ratio1*). The interval bounds are set based on the degree to which the dataset is imbalanced (179/48 ≈ 3.7) to reach a relatively balanced class distribution. 2) For undersampling, the undersampling ratio (number of undersampled majority instances/Number of majority instances) is set within the interval [0,1] (*ratio2*). Similarly, the interval bounds are set based on the degree to which the dataset is imbalanced. 3) For SMOTE, there are two related parameters, *α* and *γ*, which control the amount of oversampled minority instances and undersampled majority instances, respectively. 4) For cost-sensitive learning, the parameter *threshold* tunes the decision boundary of the classifiers. The detailed parameter settings are shown in Table 2.

We use LOOCV to evaluate how well the classifiers detect LASA cases, as the results of LOOCV are reproducible [41]. Five hundred replications are carried out for each set; the justification is given in the appendix. All the experiments are implemented in the R v3.3.1 (64-bit) platform using the “MASS,” “e1071,” “cvTools,” “plyr,” “DMwR,” and “tree” packages [42].

### 3.4. Performance Evaluation Criteria

Appropriate evaluation criteria are crucial for assessing the binary classification performance of the methods. Common evaluation criteria include accuracy, recall, precision, specificity, and so on As the minority class may bias the decision boundary and has little impact on accuracy [40], we focus on performance evaluation metrics recall, precision, *F*-score, and specificity. The confusion matrix is given in Table 3.

(5) $$\begin{array}{c} Precision=\frac{TP}{TP+FP},\\ Recall=\frac{TP}{TP+FN},\\ F‐score=2\cdot \frac{precision\;recall}{precision+recall},\\ Specificity=\frac{TN}{FN+TN}. \end{array}$$

In assessing information retrieval, recall denotes the percentage of retrieved objects that are relevant; in the context of imbalanced classification, that is the percentage of correctly classified minority instances. Precision denotes the percentage of relevant objects that are identified for retrieval. *F*-score represents a harmonic mean between recall and precision. Specificity denotes the percentage of correctly classified majority instances. In many detection tasks, recall is the primary measure, as identifying rare but significant cases in massive unstructured healthcare datasets is our major concern. As there is always a tradeoff between recall and specificity, indiscriminately improving recall can result in a significant amount of false alarms, reflected in low specificity scores and poor overall classification accuracy. Therefore, in this study, the overall classification accuracy is controlled by specifying accuracy above 80% to avoid bias when applying rebalancing strategies.

## 4. Results

### 4.1. Selection of Base Classifier

As shown in Table 4, all the conventional classifiers achieve good overall classification accuracy (above 80%). Among these classifiers, LR performs best in detecting LASA cases (recall = 0.521), which are our cases of interest. LR is also superior to other classifiers in terms of the synthesized measure (*F* − score = 0.595). These results are consistent with the conclusion in [5]. We thus select LR as the base classifier. However, it should be noted that the capability of LR for detecting LRSA cases is still unsatisfactory, due to the challenges associated with an imbalanced dataset.

### 4.2. Performance of Classifiers with Different Rebalancing Strategies

In this subsection, we investigate the effectiveness of the proposed rebalancing strategies. As described in the previous subsection, we adopt LR as the base classifier.

#### 4.2.1. Experiment Results: Data-Level Approach

**Figure 3:**
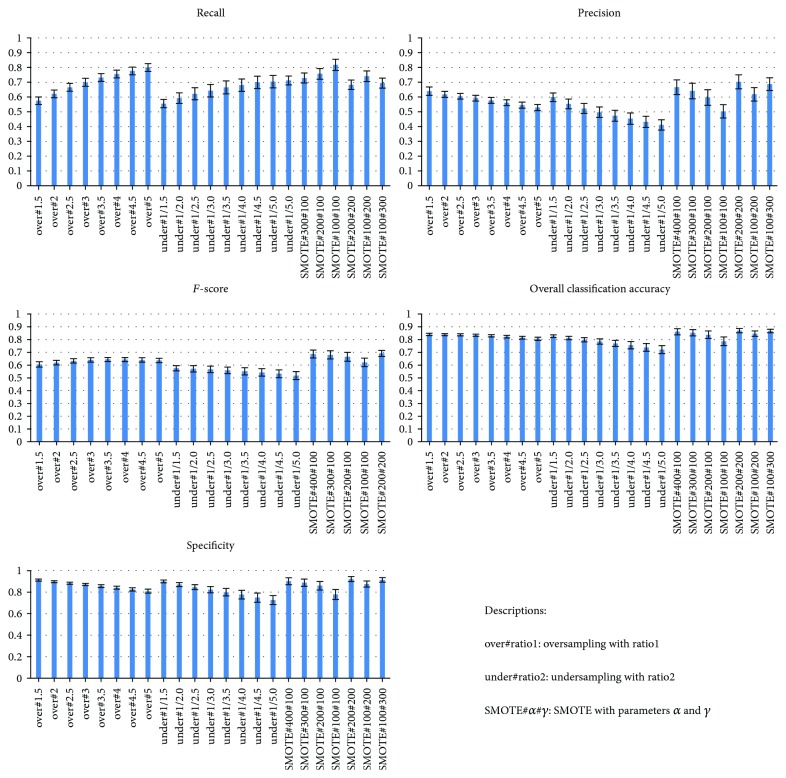
Comparison of data-level approaches.

We examine the effectiveness of combining LR with various rebalancing strategies.  Figure 3 compares the effectiveness of different data-level approaches for detecting LASA cases. As LASA cases are the minority class in the target dataset, the recall value indicates the method's accuracy. As shown in Figure 3, recall increases as the oversampling or undersampling ratio increases. In other words, the accuracy in the detection of LASA cases can be improved by making the class distribution more balanced, either by enlarging the size of the minority class or reducing the size of the majority class. However, there is always a tradeoff between recall and specificity/precision; the specificity/precision decreases as the recall grows. Compared with LR alone, LR in conjunction with resampling strategies gives a superior performance.

We then compare the classifiers with the best performance, all of which achieve an overall classification accuracy within the interval [0.82, 0.85], as shown in Table 5. Specifically, LR combined with oversampling improves the recall and *F*-score by 40.50 and 8.40%, respectively, under the setting *ratio*1 = 3.5; LR combined with undersampling improves the recall by 6.53% and decreases the *F*-score by 3.36%, respectively, under the setting *ratio*2 = 1/1.5; and LR combined with SMOTE outperforms all of the classifiers, improving recall and *F*-score by 45.30% and 11.76%, respectively, under the settings *α* = 200 and *γ* = 100. As can be seen from the results, recall can be significantly improved with only a slight sacrifice (around 1.5%) in overall classification accuracy.

**Figure 4:**
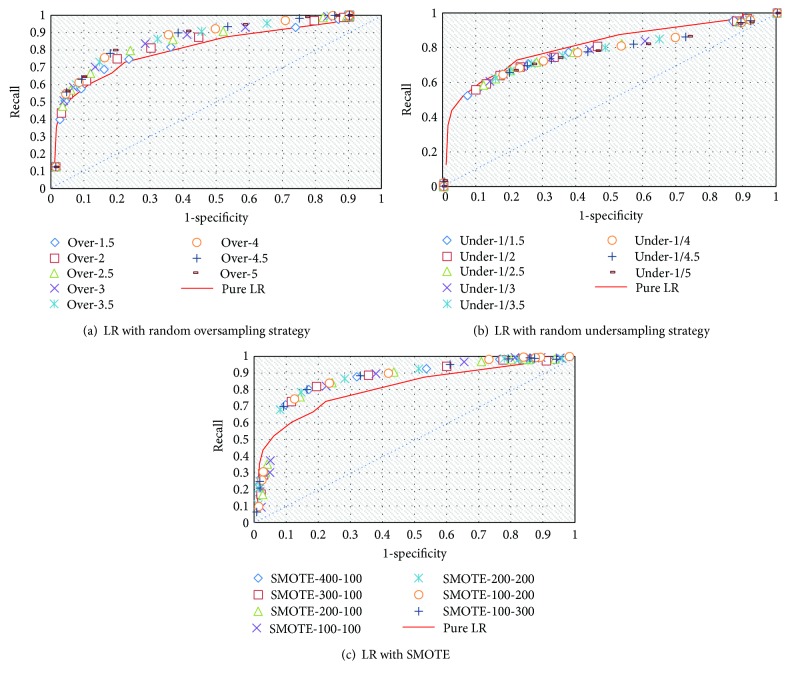
ROC curves of LR with different resampling strategies.

To evaluate the robustness of the three approaches, we plot their receiver-operating characteristic (ROC) curves, that is, true positive rate (sensitivity) against false positive rate (1 − specificity), as shown in Figure 4. The blue dashed line in each plot describes the performance of a “completely random guess” for the class of observation (i.e., the no-discrimination line from coordinates (0,0) to (1,1)), and the red line describes the ROC curve of the base classifier (pure logistic regression) for comparison purposes. A good classification method should yield points in the upper region or near the coordinate (0,1). As shown in Figure 4, all of the plots of the true positive rate against the false positive rate are above the no-discrimination line, indicating that LR combined with resampling strategies effectively reduces the effects of the two-class classification problem. The ROC curve of LR combined with SMOTE is closer to coordinate (0,1) than the ROC curve of the pure LR, indicating its superior ability to detect LASA cases.

#### 4.2.2. Experiment Results: Algorithm-Level Approach

**Figure 5:**
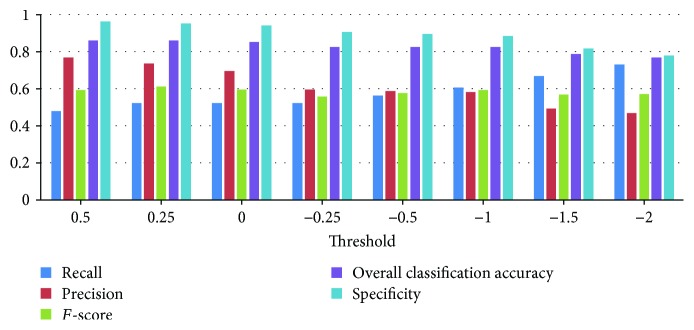
Comparison of cost-sensitive learning methods with various thresholds.

We also apply the cost-sensitive learning method to the detection of LASA cases. Again, LR is used as a base classifier.  Figure 5 shows the classification results under various parameter settings. A smaller threshold value indicates that the decision boundary is more biased toward the majority class, that is, the non-LASA class, which increases the probability that an unknown case will be identified as a LASA case. As can be seen from Figure 5, as the threshold approaches the majority class, the recall increases and the precision decreases. The algorithm achieves the best performance when the threshold is −1; at this level, the recall and *F*-score are 14.21% and 2.93% higher, respectively, than when only the base classifier is used.

## 5. Discussion

### 5.1. Key Findings

In this study, we develop a framework for analyzing imbalanced data using rebalancing strategies. We test the effectiveness of various rebalancing strategies on a medical incident reports dataset. We conduct a comparative analysis of techniques for automatically detecting LASA cases (an imbalanced classification problem) using classifiers combined with different rebalancing strategies, including both data- and algorithm-level approaches. As there is always a tradeoff between recall and specificity, indiscriminately improving recall can result in a significant number of false alarms, reflected in low specificity and poor overall classification accuracy. The methods developed in this study maintain the overall classification accuracy at an acceptable level (accuracy > 80%) by applying rebalancing strategies.

**Figure 6:**
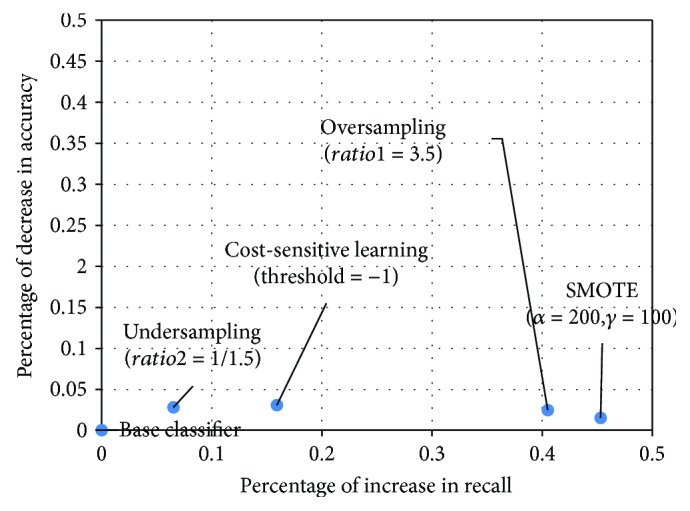
Summary of changes in recall and accuracy for difference classifiers.

The results show that data-level approaches, including oversampling, undersampling, and SMOTE, are better for detecting LASA cases than algorithm-level approaches, perhaps due to the uncertainty and inconsistency of the cost matrix in training and testing the dataset. Among the data-level approaches, combining the base classifier with SMOTE, achieves the best performance; it improves the detection accuracy of LASA cases by 43.2% compared with the base classifier alone, without much loss of overall classification accuracy. There are two explanations for this result. (1) As discussed in [11], an unavoidable consequence of undersampling is a loss of information. As our dataset contains only 227 cases, randomly undersampling the majority class can result in incomplete information, which affects decision boundary learning. This explains why a random oversampling strategy generally performs better than a random undersampling strategy on small datasets. (2) Oversampling through the random replication of the minority class sample usually creates very specific rules, leading to model overfitting [7]. With replication, the decision region for the minority class can become smaller and more specific. In contrast, SMOTE builds larger decision regions that contain nearby minority class points, resulting in a higher recall.  Figure 6 summarizes the changes in accuracy and recall of the different classifiers with their best performance. Taking the base classifier as a reference, it can be observed that in all the tested rebalancing strategies, the increase in recall is significantly higher than the decrease in overall detection accuracy. Specifically, the base classifier combined with SMOTE achieves the greatest increase in recall (45.3%) and the smallest decrease (1.5%) in accuracy.

It should be noted that there is no universal solution for all problems. Although SMOTE outperforms other rebalancing strategies for our dataset, it may have a higher computation cost. Due to the considerable growth in the size of the training dataset caused by the addition of synthetic samples, the training time for the resulting balanced data would be relatively higher than that for the original data. Again, as the size of our dataset is not massive, the increased computation cost is negligible. However, the overall decision-making procedure would be time costly for datasets with huge sizes and high dimensionality. In addition, algorithm-level approaches such as the cost-sensitive method may outperform data-level approaches if the cost matrices for training and testing the data are empirically known. It is important to evaluate the performance of different rebalancing strategies under the condition of no prior knowledge of data scale and cost matrices.

Nowadays, more and more crowdsourced medical data are becoming available due to the fast growth in health-related applications. As a result, techniques for analyzing big health data offer a promising and practical research direction [43]. Detecting rare but significant healthcare events through statistical data analytics may reduce treatment costs, avoid preventable diseases, and improve care quality in general. One typical application is classifying medical incident reports. Classifying incident reports at a granular level, identifying specific incident reports related to major adverse events, and discovering vital information hidden in the reports are all crucially important steps for improving patient safety. In general, the overall procedure for identifying medical incident reports involves structuring the unstructured text data using text mining to extract key terms [44, 45] and then constructing classifiers on the structured dataset for detecting specific medical incident reports. However, the imbalanced data property of incident report datasets makes such detection a challenging task.

Classifying imbalanced datasets has been recognized as a common problem in healthcare data analytics applications, such as cancer diagnostics, medical incident reports detection, and disease risk prediction. Typically, in these applications, correctly detecting minority class instances is crucial, as they correspond to high-impact events. However, the imbalance property of these datasets makes this a challenging task. Most standard classifiers, such as logistic regression and support vector machine, implicitly assume the equal occurrence of both classes, and these methods are designed for maximizing the overall classification accuracy. As a result, they favor the majority class, resulting in poor sensitivity toward the minority class. The findings in this study may help improve the detection of medical incidents caused by LASA drug mix-ups in imbalanced datasets and may eventually eliminate the need for manual identification of similar mediation-related harmful incidents. It is worth noting that the rebalancing strategies discussed in this study are not limited to medical incident report detection; they have a broad range of applications involving classification of datasets with similar imbalanced data properties.

### 5.2. Future Directions

In this study, we examine a two-class imbalanced classification problem using a simple illustrative example. The developed framework is potentially useful for multiclass classification problems, that is, when there are multiple classes of unequally distributed size. However, it would be difficult to learn the informed boundaries between classes in this scenario, as one has to find equilibrium between class size and detection significance when implementing rebalancing strategies. In the future, we will investigate the multiclass imbalanced classification problem in healthcare data analytics applications, such as the automatic detection of multiple types of medical incident reports. We also plan to develop an R package incorporating the imbalanced classification framework, which should considerably benefit both researchers and practitioners faced with the imbalanced classification problem in healthcare data analytics.

## 6. Conclusion

Detecting rare but significant healthcare events in massive unstructured datasets is now a common task in healthcare data analytics. This study is the first systematic attempt to identify rare events in unstructured healthcare datasets that have imbalanced distribution. We develop a classification framework that incorporates various rebalancing strategies for healthcare data analytics and provide some guidelines for tackling similar problems.

## Figures and Tables

**Figure 7:**
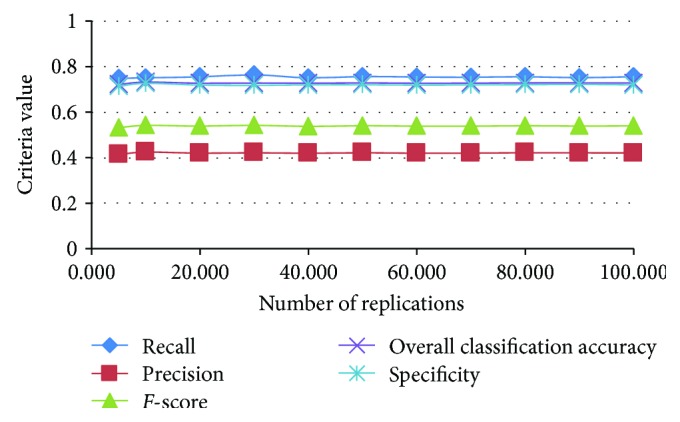
Performance evaluation of increasing numbers of replications.

**Figure 8:**
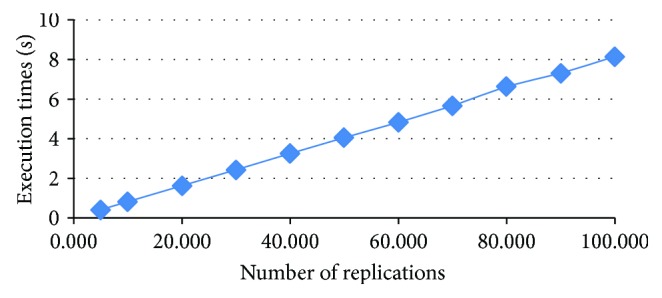
Execution time versus replications.

**Table 1:** Cost matrix.

	Prediction		
Actual		Positive	Negative
	Positive	0	C(FN)
	Negative	C(FP)	0

**(a):** 

A.1 Random oversampling + LR								
Settings for *ratio1*								
*ratio1*	1.5	2.0	2.5	3.0	3.5	4.0	4.5	5.0
A.2 Random undersampling + LR								
Settings for *ratio2*								
*ratio2*	1/1.5	1/2.0	1/2.5	1/3.0	1/3.5	1/4.0	1/4.5	1/5.0

**(b):** 

A.3 SMOTE + LR							
Settings for *α* and *γ*							
*α*	400	300	200	100	200	100	100
*γ*	100	100	100	100	200	200	300

**(c):** 

A.4 Cost-sensitive learning + LR								
Settings for *threshold*								
*threshold*	0.5	0.25	0	−0.25	−0.5	−1	−1.5	−2

**Table 3:** Confusion matrix.

	Condition positive	Condition negative
Test outcome positive	True positive (TP)	False positive (FP)
Test outcome negative	False negative (FN)	True negative (TN)

**Table 4:** Performance of conventional classifiers.

	LR	L.SVM	DT	R.SVM
Recall	0.521	0.479	0.375	0.396
Precision	0.694	0.767	0.750	0.792
*F*-score	0.595	0.590	0.500	0.528
Accuracy	0.850	0.859	0.841	0.850

**Table 5:** Comparison of classifiers with the best performance.

	Base classifier	Oversampling *ratio*1 = 3.5 (% increase over LR)	Undersampling *ratio* 2 = 1/1.5 (%increase over LR)	SMOTE *α* = 200, *γ* = 100 (% increase over LR)
Recall	0.521	0.732 (40.50%)	0.555 (6.53%)	0.757 (45.30%)
Precision	0.694	0.577 (−16.86%)	0.598 (−13.83%)	0.597 (−13.98%)
*F*-score	0.595	0.645 (8.40%)	0.575 (−3.36%)	0.665 (11.76%)
Overall classification accuracy	0.850	0.829 (−2.47%)	0.826 (−2.82%)	0.837 (−1.53%)

## Data Availability

All of the datasets used in this study are publicly available at the Global Patient Safety Alerts (GPSA) system.

## Appendix

### Parameter Settings for Algorithms

The sampling schemes should be repeated to obtain unbiased evaluation results. To estimate the least number of sampling replications necessary (*rep*) and assess the experiment scale, we execute the algorithm “Random oversampling + LR” (*ratio*1 = 5) with an increasing number of replications.  Figure 7 displays the averaged evaluation result with an increasing number of replications. As can be seen, the result tends to be stable when the number of replications approaches 100. It is worth noting that the execution time grows linearly as the replication increases, which can be observed in Figure 8. In the implementation, the number of replications is fixed at 500 to ensure a reliable evaluation.

## Abbreviations

LASA: Look-alike sound-alike
LOOCV: Leave-one-out cross-validation
SMOTE: Synthetic minority oversampling technique
FN: False negative
FP: False positive
LR: Logistic regression
L.SVM: Support vector machine with linear kernel
R.SVM: Support vector machine with radial kernel
DT: Decision tree
ROC: Receiver operating characteristic
EHRs: Electronic health records.
